# Functionalised Mesoporous Silica Thin Films as ROS-Generating Antimicrobial Coatings

**DOI:** 10.3390/ijms26157154

**Published:** 2025-07-24

**Authors:** Magdalena Laskowska, Paweł Kowalczyk, Agnieszka Karczmarska, Katarzyna Pogoda, Maciej Zubko, Łukasz Laskowski

**Affiliations:** 1 Institute of Nuclear Physics Polish Academy of Sciences, PL-31342 Krakow, Poland; agnieszka.karczmarska@ifj.edu.pl (A.K.); katarzyna.pogoda@ifj.edu.pl (K.P.); 2 Department of Animal Nutrition, The Kielanowski Institute of Animal Physiology and Nutrition, Polish Academy of Sciences, 05-110 Jabłonna, Poland; p.kowalczyk@ifzz.pl; 3 Institute of Materials Engineering, University of Silesia in Katowice, 75 Pułku Piechoty 1A St., 41-500 Chorzów, Poland; maciej.zubko@us.edu.pl

**Keywords:** ROS, single-atom catalysis, antibacterial coatings, functional materials, SBA-15 silica, thin films

## Abstract

The recent COVID-19 pandemic has made the public aware of the importance of combating pathogenic microorganisms before they enter the human body. This growing threat from microorganisms prompted us to conduct research into a new type of coating that would be an alternative to the continuous disinfection of touch surfaces. Our goal was to design, synthesise and thoroughly characterise such a coating. In this work, we present a nanocomposite material composed of a thin-layer mesoporous SBA-15 silica matrix containing copper phosphonate groups, which act as catalytic centres responsible for the generation of reactive oxygen species (ROS). In order to verify the structure of the material, including its molecular structure, microscopic observations and Raman spectroscopy were performed. The generation of ROS was confirmed by fluorescence microscopy analysis using a fluorogenic probe. The antimicrobial activity was tested against a wide spectrum of Gram-positive and Gram-negative bacteria, while cytotoxicity was tested on BALB/c3T3 mouse fibroblast cells and HeLa cells. The studies fully confirmed the expected structure of the obtained material, its antimicrobial activity, and the absence of cytotoxicity towards fibroblast cells. The results obtained confirmed the high application potential of the tested nanocomposite coating.

## 1. Introduction

Microbial infections, leading to sepsis and septic shock, among other things, are one of the main causes of death and permanent health impairment in patients [1,2,3]. Rapid population growth promotes the formation of human clusters, which in turn increases the risk of transmission of dangerous pathogens [4,5]. The recent COVID-19 pandemic has clearly highlighted the threat posed by microorganisms [6]. At the same time, it has highlighted the importance of neutralising pathogens before they enter the body [7]. In the case of the SARS-CoV-2 coronavirus, its ability to infect surfaces outside the human body can persist for up to three days [8,9]. The situation is even more serious for pathogenic bacteria and yeasts. For example, fungi of the genus *Candida* can survive for up to five months on smooth surfaces such as plastic or steel [10,11,12], while the most common bacteria (*Staphylococcus aureus*, *Clostridioides difficile*, *Escherichia coli*, *Acinetobacter baumannii*, or *Mycobacterium tuberculosis*) can survive for up to six months [10,13].

For this reason, disinfecting touch surfaces is so important, especially in healthcare facilities, where harmful microorganisms are common. However, the continuous application of disinfectants requires enormous resources and is often impossible for technical reasons (operating theatres during procedures or crowded waiting rooms in healthcare facilities). Therefore, there has recently been intensified scientific research into antimicrobial surfaces that could combat or prevent the growth of harmful microorganisms without human intervention.

As shown in [14], the laser texturing of surfaces to obtain a shark skin-inspired structure damages bacterial cell membranes and reduces bacterial adhesion by 94% compared to a flat surface. Although such a surface does not require chemical modification and exhibits durability due to the incorporation of the structure into the material, it is difficult to produce, has limited effectiveness (practical reduction ∼90–95%), and lacks biocidal activity (only delays colonisation). Superhydrophobic coatings are an interesting solution. As demonstrated in [15,16], coatings made of saturated fatty acids have a wetting angle of ∼165°, are non-toxic, and combat *E. coli* and *Listeria*. Such coatings are biocompatible, easy to manufacture, and environmentally friendly. However, they are susceptible to mechanical degradation, have limited effectiveness against various pathogens, and lack toxic effects on microorganisms. Chitosan coatings work on a similar principle [17,18,19], as do silica-based mesoporous nanostructures [20]. In this case, their action is also based on preventing bacteria from adhering to surfaces. Unfortunately, in this case too, we cannot talk about the elimination of microorganisms: we can talk only about the prevention of their growth.

The active elimination of microorganisms is observed, however, for coatings made of heavy metals such as silver, copper, or gold [21,22]. In this case, high microbial elimination efficiency of up to 99% is observed [22]. In addition, a synergistic effect between different metals can be achieved, increasing the effectiveness [23]. However, in this case, several significant disadvantages of such coatings are observed. These include susceptibility to corrosion and abrasion, high layer weight and manufacturing cost, the release of ions into the environment, and limited duration of action, especially in the case of silver [24,25,26,27]. This problem can be somewhat reduced by encapsulating the active ingredient inside an inert matrix such as porous silica [28,29]. In this case, the silver or thymus essential oil is released gradually, significantly extending the life of the material. However, as can easily be seen, although the duration of effective action will be prolonged, the effect of such a material will diminish over time.

Research on zinc oxide coatings, both as thin films and nanoparticle-based layers, is extremely promising. Thin ZnO films (∼313 nm) deposited via atomic layer deposition (ALD) on glass and PPE fabrics have demonstrated virtually 100% elimination of *E. coli*, effectively preventing biofilm formation; however, the ALD process lacks practicality for large-scale production [30]. Similarly, coatings formed by dispersing ZnO nanoparticles on surfaces are highly effective [31], but they suffer from issues such as an uneven distribution of particles and vulnerability to surface contamination. The antimicrobial performance of ZnO is predominantly attributed to the generation of reactive oxygen species (ROS) [32,33], yet the cytotoxicity of ROS to mammalian cells remains a concern [34], and their production often requires external activation such as light or ultrasound [35,36].

In the publication [37], it was demonstrated that optimising the morphology of ZnO nanoparticles in hybrid ZnO/graphene oxide/montmorillonite coatings improves both barrier and antibacterial properties while maintaining film integrity and reducing Zn^2+^ ion release. In the study [38], a poly(allylamine hydrochloride) (PAH)/ZnO multilayer coating was applied to textile substrates, achieving over 99% bacterial reduction with nanoparticle release below 30 μg ${\mathrm{cm}}^{-2}$, remaining well under cytotoxicity thresholds. In the work [39], green-synthesised ZnO coatings deposited on cotton fabrics provided effective UV protection and strong antibacterial performance, although some limitations in coating adhesion and long-term durability under washing cycles were observed.

These recent approaches address key limitations of earlier ZnO coatings by improving nanoparticle stabilisation, biocompatibility, and functional longevity. However, uniform distribution, mechanical robustness under real-world conditions, and sustained ROS generation without external stimuli remain critical challenges limiting widespread application.

Looking at the above literature review, ROS-generating coatings appear to be the most promising. For these reasons, we decided to design a nanocomposite coating that is capable of generating, storing, and then releasing ROS to eliminate harmful microorganisms, prevent biofilm formation, and reduce microorganism survival. However, such a material should be free from the disadvantages of the previously presented materials. Our assumption was that the material should be able to produce reactive oxygen species without the need for external stimulants such as light or sound. For this, the presence of moisture should be sufficient. We assumed an ROS generation mechanism similar to that of single-atom catalysts. This will allow control of the ROS generation mechanism and their type. However, this requires very precise control of both the configuration of the active catalytic groups and their relative positions to avoid several different ROS generation mechanisms that may change, for example, in the presence of an external factor. With defined catalytic centres positioned at a specific distance from each other, single-atom catalysis will dominate instead of, for example, a mechanism requiring bond conversion [40,41,42,43].

Using molecular engineering methods, we have precisely designed such a material and prepared an appropriate synthesis procedure to obtain the desired structure. The designed material consists of two components. The first is a porous silica matrix responsible for the durability of the coating, accumulation and release of ROS, and the second component is appropriately selected and separated functional groups responsible for the generation of ROS. The functional groups are appropriately distributed within the matrix. We chose mesoporous silica SBA-15 as the matrix, which seems ideal for such solutions. It is chemically and thermally stable over a wide range of pH and temperatures [44,45,46]. It has a large specific surface area (up to 1000 m^2^/g), which can accommodate a huge number of functional groups (which affects the catalytic activity of the target material), and a significant pore volume (over 1 cm^3^/g), providing space for the generation and accumulation of ROS. Micro- and mesoporosity ensure the smooth release of ROS into the environment [47,48]. In addition, SBA-15 silica coating techniques are efficient, inexpensive, and relatively fast. They can be applied to substrates made of various materials and with irregular shapes. While other mesoporous silicas such as MCM-41 or KIT-6 also offer high surface areas and tunable pore architectures, SBA-15 provides a unique combination of advantages that are particularly suitable for our application. Compared to MCM-41, SBA-15 possesses larger and thicker pore walls, which improves thermal and hydrothermal stability [45,49]. In contrast to KIT-6, SBA-15 exhibits a simpler 2D hexagonal pore arrangement, facilitating more controlled functionalisation and diffusion dynamics. Moreover, unlike amorphous or disordered mesoporous silicas, SBA-15 offers superior structural regularity and reproducibility in synthesis, which is essential for ensuring consistency in ROS-related functional behaviour. These factors, along with the proven compatibility of SBA-15 with surface modification methods, established procedures for functional group incorporation and effective control over their spatial distribution, make it a robust and efficient choice for designing stimulus-responsive ROS-generating coatings.

The important function of the silica matrix is to immobilise the relevant functional groups inside the pores and keep them at a certain distance from each other. This distance should be large enough to prevent them from interacting with each other (which can result in catalysis associated with bond conversion [41]) but at the same time optimally small so that their density is as high as possible (which results in high catalytic activity). The type of functional groups is crucial for the ROS generation process. We chose propyl-copper-phosphonate groups, which are known for their effectiveness in the catalytic generation of reactive oxygen species [50,51]. At the same time, they can be effectively incorporated into the structure of SBA-15 silica while maintaining full control over their distribution [52,53,54].

**Figure 1:**
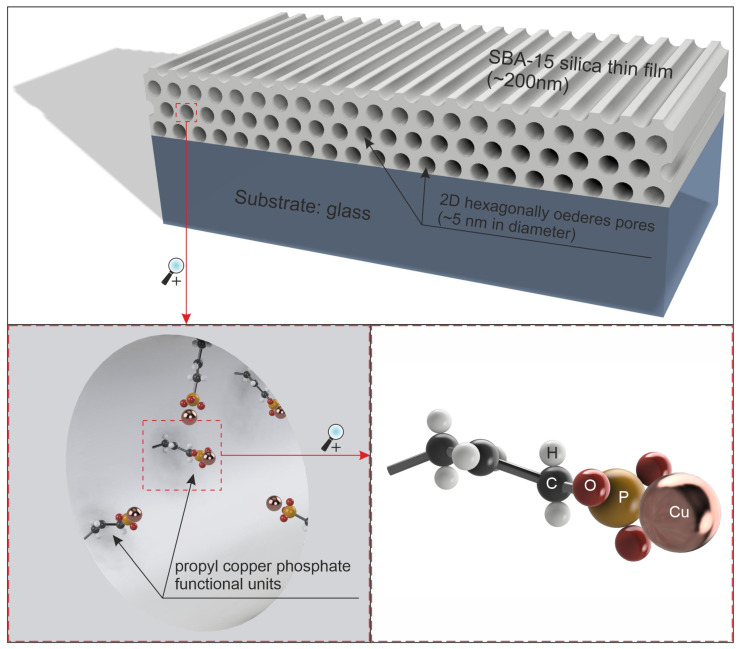
Schematic representation of ROS-generating nanocomposite layer: SBA-15 mesoporous silica thin films with propyl-copper-phosphonate groups located inside pores.

The composite material presented here can be prepared in the form of a thin layer (approximately 200 nm in thickness) applied to a suitable substrate (e.g., glass or a metal surface) with 2D hexagonally arranged pores (with a diameter of approximately 5 nm). Its diagram is shown in Figure 1.

This work demonstrates the efficiency of the proposed coating in bacteria elimination and the influence of the concentration of propyl-copper phosphonate functional groups on the antimicrobial properties of the nanocomposite. Therefore, we have prepared coatings with four different concentrations of functional propyl-copper-phosphonate groups to test the effect of functionalisation rate on antimicrobial properties of the nanocomposite. The ability to control the degree of cytotoxicity of the coating against dangerous microorganisms could be an excellent solution for food packaging. In particular, in packaging for yoghurt or other products containing bacteria, it is desirable to control the growth of microorganisms rather than completely eliminate them. Therefore, studies on the influence of functional group concentrations on antimicrobial properties will not only allow the most effective configuration to be identified but will also enable the selection of concentrations that are suitable for stabilising the quantity of microorganisms on their surface.

For the synthesis, we used the combination of the evaporation-induced self-assembly (EISA) method with co-condensation, which gives excellent results in terms of the uniformity of functional group distribution and precise control of their concentration. This method allows overcoming one of the biggest problems in the functionalisation of silica structures: namely, pore blocking. This problem is particularly relevant in functionalisation using larger systems and may lead to incomplete functionalisation and a lack of control over the functional group concentration. For the study, we prepared materials with the assumed concentration of functional groups (in moles, relative to SiO_2_ groups) of 1.25, 2.5, 5, and 10%. In the rest of the text, we refer to them as MSTF-CuX, where X denotes the assumed concentration of functional groups.

## 2. Results and Discusion

### 2.1. Physico-Chemical Analysis

The obtained thin layers of mesoporous silica were examined to verify their chemical composition and molecular structure. The research methodology was selected to confirm the obtained structure with evenly distributed copper ions inside the silica matrix. Due to the fact that intermolecular interactions resulting from the concentration of functional groups inside the silica pores are a particularly important factor influencing the properties of the nanocomposite material, it was necessary to check whether the material obtained had the assumed degree of functionalisation (which entails a specific distribution of functional groups) and to check whether the change in the degree of functionalisation was monotonic and proportional to the changes in the precursors of functional groups at the synthesis stage (which entails the possibility of controlling the distribution of functional groups).

Thin SBA-15 silica films obtained via dip-coating are characterised by the presence of long cylindrical mesopores parallel to the substrate with a 2D hexagonal spatial arrangement and microporosity typical of SBA-15.

**Figure 2:**
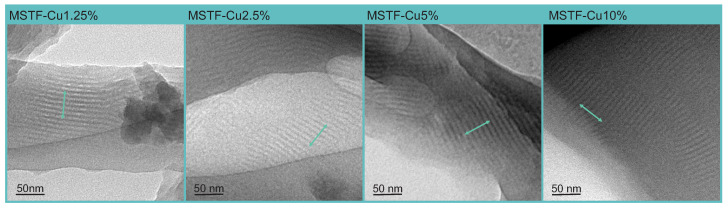
Transmission electron microscopy images of obtained samples: mesoporous silica thin films containing various concentrations of propyl-copper-phosphonate functional units inside pores. The green markers on the TEM micrographs are 50 nm long and are arranged perpendicular to the pore length to enable the evaluation of the structural dimensions of the samples.

A detailed analysis of TEM images confirmed all assumed structural parameters of the silica coating. Figure 2 shows samples with all assumed functional group concentrations and characteristics typical of a 2D hexagonally ordered SBA-15 structure. Namely, we can observe long, parallel pores with a constant network (defined here as the smallest distance between the centres of the pores in the direction perpendicular to their length) of approximately 8.5 nm and pores with a diameter of approximately 5 nm. No structural distortions, impurities, or crystallites are visible. This means that the co-condensation method used to introduce functional groups into the silica structure does not adversely affect the condensation process and the final pore arrangement. This test was important because obtaining an undisturbed SBA-15 structure determines the biological activity of the material, as it ensures adequate space for the accumulation of ROS and channels for their release.

With the assumed structure of the silica matrix confirmed, the key aspect remains to verify the assumed concentration of functional groups and, consequently, their distribution. In order to maintain the correct stoichiometry, appropriate proportions between the silica precursors (tetraethyl orthosilicate—TEOS) and functional groups (phosphonate propyl dietyltriethoxysilane—PPTES) were assumed during the synthesis stage, theoretically allowing for the achievement of predetermined Si:P and Si:Cu ratios in the target material. For example, the MSTF-Cu10% sample, containing the highest assumed concentration of functional groups (10%), was synthesised assuming a TEOS:PPTES ratio of 9:1, which theoretically allows for a Si:P ratio of 10:1 in the target material (it should be remembered that PPTES contains one silicon atom). Importantly, the P:Cu ratio should remain constant at 1:1 in all samples, as each -POO_2_Cu functional group contains one copper atom and one phosphorus atom.

**Figure 3:**
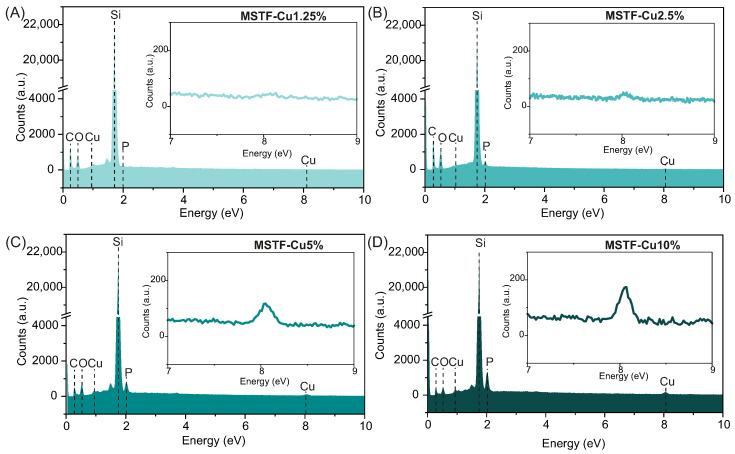
Energy-dispersive X-ray spectra of (**A**) MSTF-Cu1.25%, (**B**) MSTF-Cu2.5%, (**C**) MSTF-Cu5%, and (**D**) MSTF-Cu10%. Insets show magnified views of the 7–9 keV region, highlighting the Cu Kα peak.

To confirm the incorporation of the assumed amounts of copper and phosphorus into the silica structure, energy-dispersive X-ray spectroscopy (EDS) was performed in combination with scanning electron microscopy (SEM), analysing the proportions between the key elements, i.e., silicon, phosphorus and copper. The EDS spectra can be seen in Figure 3, while results of the elemental analysis, including the atomic ratios of Si:P and P:Cu, are summarised in Table 1.

The energy-dispersive X-ray spectra (Figure 3) clearly exhibit characteristic peaks corresponding to all expected elements: Si Kα (1.74 keV), P Kα (2.01 keV), and Cu Kα (8.05 keV), in addition to carbon and oxygen signals. A progressive increase in the intensities of the phosphorus and copper peaks with increasing nominal precursor concentrations qualitatively confirms successful control over the synthesis process. The EDS analysis revealed systematic trends that correlated well with the intended functional group loadings. Specifically, the silicon content decreased from 98.9% in the MSTF-Cu1.25% sample to 91.2% in MSTF-Cu10%, while the phosphorus content increased proportionally from 1.1% to 8.8%. This inverse relationship supports the successful incorporation of phosphonate groups in accordance with the designed stoichiometry. A slight deviation from the assumed content of 10% for the MSTF-Cu10% sample can be attributed to analytical uncertainty associated with the detection of light elements in EDS analysis. The measured atomic ratios of P:Cu were close to 1:1; however, a decreasing trend in copper content is clearly visible as the concentration of functional groups in the material increases. The changes are small but clearly visible. As we observed for powder materials, the configuration of copper coordination changes with the concentration of functional groups. At low functional group concentrations, copper ions are predominantly coordinated by single anchoring groups (article in preparation). In contrast, at high concentrations (e.g., 20%), the dominant configuration involves the coordination of a single copper ion by two anchoring groups, resulting in a Cu:P ratio of approximately 1:2 [52]. It is important to note that the transition between these two coordination regimes is not necessarily abrupt. Rather, the distribution between mono- and bidentate coordination likely shifts gradually as the functional group concentration increases. Consequently, an increase in the number of anchoring groups may lead to a lower overall copper content, due to a higher proportion of bidentate (1:2) coordination configurations, which is probably what we observe in the materials studied here.

The results of the study significantly confirm the compliance of the synthesis results with the assumptions.

**Figure 4:**
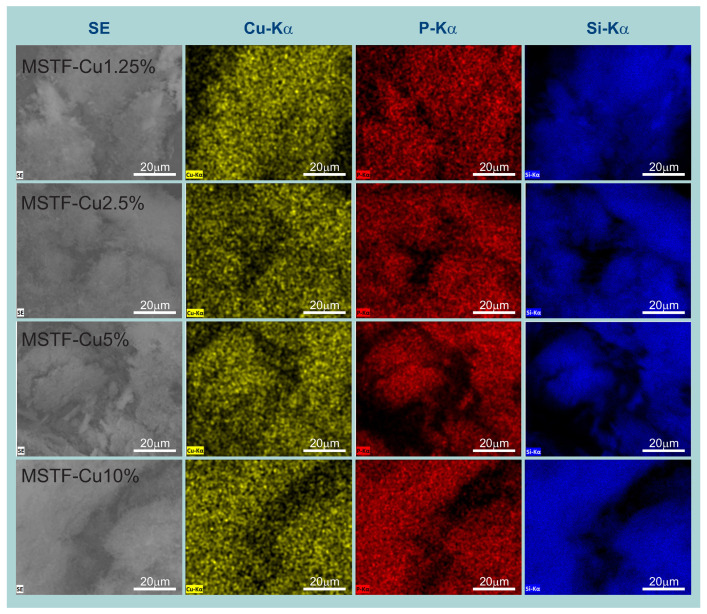
SEM images juxtaposed with EDS elemental mapping of investigated samples: mesoporous silica thin films containing various concentrations of propyl-copper-phosphonate functional units inside pores.

EDS elemental mapping provided crucial insights regarding spatial distribution homogeneity across sample areas. Large-area elemental maps for Si, P, and Cu (Figure 4) demonstrated the uniform distribution of all elements throughout the coating thickness, confirming the successful homogeneous incorporation of functional groups within the silica matrix. Furthermore, the overlapping spatial distribution of phosphorus and copper signals confirms the presence of intact copper phosphonate complexes rather than phase-separated copper species.

**Figure 5:**
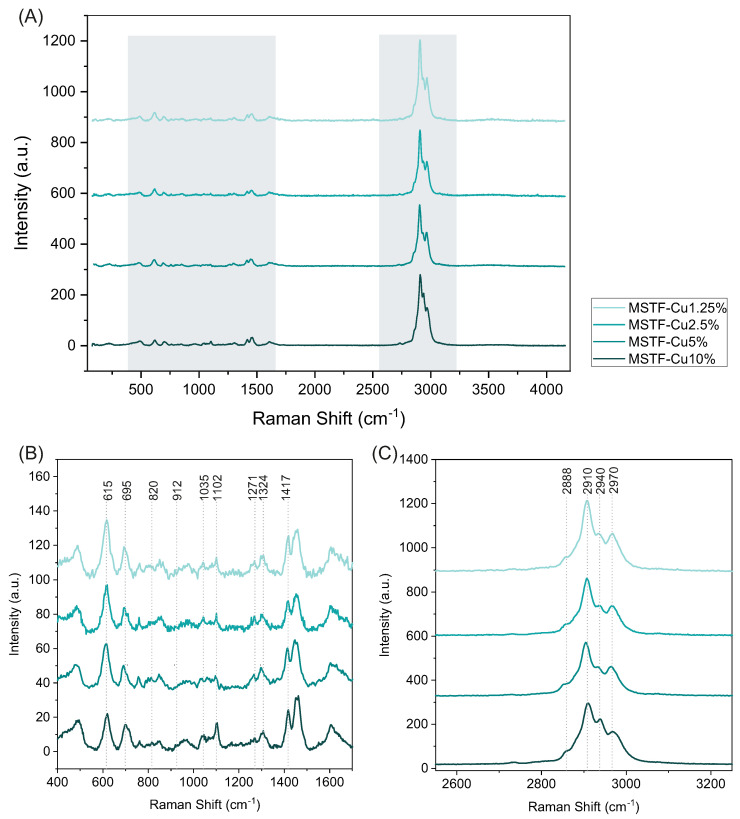
Raman spectra obtained for samples tested with labeled characteristic bands: full range (**A**), selected area from 400 to 1700 ${\mathrm{cm}}^{-1}$ (**B**), selected area from 2550 to 3250 ${\mathrm{cm}}^{-1}$ (**C**).

Another important factor influencing the properties of the obtained nanocomposite is its molecular structure—particularly that of the functional groups. To confirm the proposed molecular structure of the silica functionalised with propyl-copper-phosphonate moieties, Raman spectroscopy was performed. In Figure 5, the Raman spectra of four samples with varying concentrations of these groups (from 1.25% to 10%) are presented. A comprehensive analysis of the obtained spectra was carried out, building upon our previous studies in which we employed density functional theory (DFT) simulations and carefully designed silica models functionalised with propyl-copper-phosphonate groups. These prior efforts enabled us to confidently assign spectral bands characteristic of the target functional groups [52,53,55,56,57].

In this study, particular attention was paid to the positions and intensities of bands corresponding to vibrational modes associated with CH_2_, Si–O–C, O–P–O, and O–Cu–O groups. According to the synthesis design, the prepared samples contained 10%, 5%, 2.5%, and 1.25% of the propyl-copper-phosphonate functional units. This variation correlates with an inverse gradient in the amount of trimethylsilyl (TMS) groups—i.e., the sample with the highest concentration of propyl-copper-phosphonate groups is expected to contain the lowest amount of TMS groups and vice versa (see synthesis procedure in Section 3.1). Following a careful analysis of all sample spectra [58,59,60], two spectral regions containing characteristic bands were selected for detailed examination. Figure 5A presents the full-range spectra of the analysed samples, while Figure 5B,C focus on the 450–1500 cm^−1^ and 2700–3200 cm^−1^ regions, respectively, to facilitate a detailed analysis of intensity variations in the characteristic peaks.

The most prominent markers of the propyl-copper-phosphonate groups are bands at 820, 912, 1035, and 1324 cm^−1^, whose intensities decrease with decreasing concentrations of these groups. Based on theoretical calculations, numerical modelling, and reference sample testing, these bands have been attributed to deformation vibrations of the entire functional group containing the POO_2_Cu moiety and the propyl chain connecting it to the silica framework. These bands in the 450–1500 cm^−1^ region primarily originate from CH_2_, Si–O–C, O–P–O, and O–Cu–O vibrational modes [52].

Simultaneously, prominent bands originating from CH_3_ groups in the TMS moieties are observed at 615, 695, 1102, and 1271 cm^−1^ (Figure 5B). The intensity of these features increases as the concentration of propyl-copper-phosphonate groups decreases, indicating the dominant presence of TMS groups on the silica surface. The band at 1417 cm^−1^ is particularly noteworthy, as it originates from both CH_2_ and CH_3_ groups [52]. However, its intensity is predominantly influenced by the concentration of copper-containing functional groups and increases proportionally with their content.

Based on these spectral features, the samples can be grouped into two categories. The first group, containing 5% and 10% of functional groups, displays prominent bands attributed to the propyl-copper-phosphonate moieties and less intense TMS-related peaks at 615, 695, 1102, and 1271 cm^−1^. The second group, comprising the samples with 2.5% and 1.25% functionalisation, exhibits well-defined TMS-associated bands and a notably reduced intensity of the bands at 820, 912, 1035, 1324, and 1417 cm^−1^. This observation is fully consistent with the initial synthesis assumptions, wherein samples with a higher concentration of functional groups contain fewer trimethylsilyl groups and vice versa.

A similar trend is observed in the 2700–3200 cm^−1^ region (Figure 5C), although interpretation is somewhat complicated by overlapping CH_3_ and CH_2_ vibrational modes. Nonetheless, a clear distinction between the two groups remains evident. Four bands at 2888, 2910, 2940, and 2970 cm^−1^ are visible in all samples, but their relative intensities vary with the degree of surface functionalisation. Notably, the intensity of the 2888 and 2940 cm^−1^ bands decreases significantly with decreasing concentrations of copper-containing functional groups.

In summary, Raman spectroscopy provided significant evidence confirming the structural characteristics’ compliance with the design assumptions. However, a more comprehensive explanation of the molecular structure of functional groups requires research using synchrotron radiation, which is the subject of our other publication.

The synthesised material, with its molecular structure validated through the aforementioned characterisation techniques, was designed to function as a single-atom catalyst for reactive oxygen species (ROS) generation in biomedical applications. Specifically, thin films fabricated from this material are expected to exhibit antimicrobial properties through ROS formation within the mesoporous framework and subsequent release to the surface. Therefore, the confirmation of ROS presence within the coating represented a critical step in the research.

Fluorescence microscopy was selected as the analytical method to verify ROS generation, as it provides an unambiguous and direct confirmation of ROS presence despite its semi-quantitative nature. The detection of reactive oxygen species within the silica matrix was accomplished using a fluorogenic probe (CellROX Green Reagent) in phosphate-buffered saline (PBS). CellROX Green exhibits weak fluorescence in its reduced state but demonstrates intense green fluorescence upon oxidation by reactive oxygen species. The fluorescent response is characterised by maximum emission at 520 nm under 485 nm excitation wavelength.

**Figure 6:**
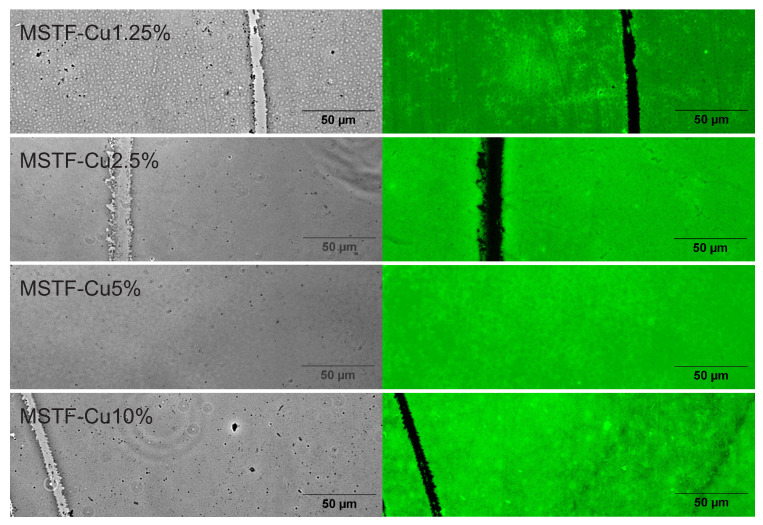
The fluorescence microscopy images of investigated samples: mesoporous silica thin films containing various concentrations of propyl-copper-phosphonate functional units inside pores.

Following probe application, fluorescence microscopy enabled the spatial localisation of ROS-containing regions. Figure 6 presents comparative bright-field and fluorescence images of the investigated samples. To emphasise the relationship between emission intensity and layer thickness, areas where the thin films exhibited partial damage were deliberately selected for analysis.

The microscopic observations confirmed the presence of reactive oxygen species in all coatings comprising porous silica matrices functionalised with copper-containing groups across the entire concentration range (1.25% to 10%). Intact thin films demonstrated uniform ROS distribution throughout their structure, as evidenced by consistent fluorescent emission. Conversely, regions devoid of composite thin films exhibited no fluorescent signal, confirming the absence of ROS in these areas and confirming the effectiveness of the detection method. It is worth noting that no significant differences were observed between the fluorescence images of the samples. However, with the equipment we used, it is not possible to quantitatively assess the generation of ROS by the tested material or to distinguish between different types of ROS. The important information is the presence of generated ROS in all tested samples.

To check the surface properties of the thin films obtained and their possible influence on the biocidal properties of the materials, we measured the wetting angle and calculated the surface free energy (SFE, $\gamma_{s}$). The obtained values were juxtaposed in Table 2, together with the SFE (last column), while photos of the droplets of the test liquids can be seen in the Supporting Information (Figure S1).

As can be seen, the SFE values do not differ significantly from one sample to another. This is in line with our expectations, as the functional groups should be located inside the pores and not on the surface of the coating (see Figure 1). The surface, by definition, should be similar for all materials tested. The result obtained confirms this and allows us to exclude the influence of surface area on the differences in the antimicrobial properties of the individual tested materials.

In order to check whether the antimicrobial properties of our materials are affected by copper released from the pores, we carried out material stability tests (copper-releasing tests; see Section 3.2). However, even after 10 days of interaction of the test samples with normal saline, no copper was recorded in the solution. Therefore, we can conclude that at a pH of approximately 6.8 (the measured pH of the normal saline used for the experiment), the material is quite stable, and it is unlikely that the biocidal properties of the films were affected by copper ions released from the material during the test. This observation is consistent with general findings observed by other researchers. Silica matrices (MSNs, SBA-15), functionalised with anchoring groups such as amino (-NH_2_), thiol (-SH), and carboxylic acid (-COOH), form stable coordination bonds with metal ions, resulting in durable immobilisation and limited metal release. Tang et al. demonstrated that bifunctionalised SBA-15 containing both –NH_2_ and –SH groups adsorbs ${\mathrm{Pb}}^{2+}$ and ${\mathrm{Cd}}^{2+}$ via chemisorption, following pseudo-second-order kinetics. The material retained its mesoporous structure and exhibited high sorption stability across a wide pH range (4–6). The metals were not desorbed under experimental conditions, confirming the selectivity and durability of the formed complexes [61]. Similar findings were reported in a review by Munaweera et al., where amino and thiol groups anchored on MSNs efficiently captured a broad range of heavy metal ions through strong coordination interactions, exceeding the sorption capacity of unmodified materials, which primarily rely on weaker physisorption mechanisms [62]. In turn, Olivieri et al. designed an advanced system in which MSNs were loaded with benzotriazole (BTA) and capped with a silver-based Ag^+^–BTA complex. This capping layer enabled pH-stimulated BTA release while simultaneously capturing Cl^−^ ions. The Ag^+^–BTA complex preserved the porosity and structural stability of the carrier and effectively prevented the uncontrolled release of metal ions, underscoring the robustness and controllability of the metal–matrix interaction [63]. This substantiates our results and allows us to conclude that the materials we obtained do not release copper into the environment and that the bacterial elimination mechanism is of a different origin.

### 2.2. Microbiological Tests

**Figure 7:**
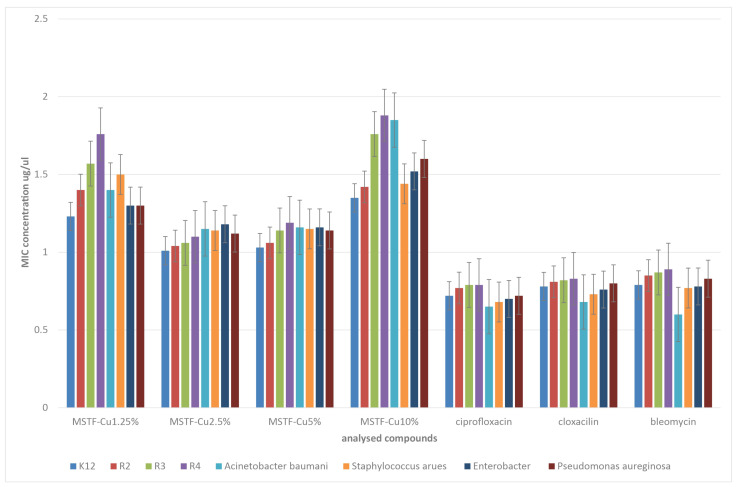
Minimum inhibitory concentration (MIC) of investigated samples: mesoporous silica thin films containing various concentrations of propyl-copper-phosphonate functional units inside pores in comparison to standard antibiotics: ciprofloxacin, cloxacillin, and bleomycin.

Microbiological tests were carried out on a wide range of pathogenic bacteria, both Gram-positive and Gram-negative: *Escherichia coli* (K12 ATCC 25404, R2 ATCC 39544, R3 ATCC 11775, and R4 ATCC 39543), *Staphylococcus aureus* strain (ATCC 23235), *Acinetobacter baumannii* (ATCC 17978), *Pseudomonas aeruginosa* (ATCC 15442), and *Enterobacter cloacae* (ATCC 49141). They were selected in such a way as to present a broad spectrum of activity of the prepared materials. The minimum inhibitory concentration (MIC) results obtained for the tested materials in comparison with reference antibiotics (ciprofloxacin, cloxacillin, and bleomycin) are shown in Figure 7. However, when comparing the activity of our materials and reference antibiotics, the method of suspension preparation should be taken into account (see Section 3.2). In the case of the tested coatings, the suspension was prepared using material scraped from 1 cm^2^ of the substrate, which, considering the layer thickness of approximately 200 nm, is an incomparably smaller amount than the mass of the antibiotic contained in the same volume of water. For technical reasons (it is not possible to weigh the scraped amount of coating), we are unable to compare the amounts of both materials. We can only say that the content of the tested materials in the suspension is at least several times lower than that of the reference antibiotics.

When analysing the results obtained for the tested coatings, a very similar trend can be clearly seen for interactions with all tested bacterial strains. Materials with all concentrations of functional groups exhibit antibacterial activity. However, this activity varies depending on the concentration of functional groups. Interestingly, this dependence is non-monotonic, which was expected. Namely, the strongest antibacterial activity is exhibited by materials with a concentration of propyl-copper-phosphonate groups of 2.5 and 5% (MSTS-Cu2.5% and MSTS-Cu5%) with their effectiveness being very similar. Materials with active group concentrations of 1.25 and 10% show significantly poorer biocidal properties with MSTS-Cu1.25% having lower MIC values than MSTS-Cu10% for all tested strains.

The observed dependence is closely related to the structure of the material and in particular to the distribution of functional groups and their distance from each other. Assuming a small distance between the functional groups, interaction between them is possible, which in the presence of water molecules may result in electron transfer and a change in the molecular configuration of these groups, combined with the generation of ROS: H_2_O_2_, hydroxyl radicals, and hydrated hydroxyl radicals. However, such a mechanism requires subsequent regeneration of the catalytic centres, so the generation of ROS is limited in time [64]. If, on the other hand, the functional groups are far enough apart that electron transfer is not possible (remember that during synthesis, surface OH groups are eliminated by sililation), the most likely outcome is the catalytic generation of other ROS: O21, $O_{2}^{\cdot -}$, $HO^{\cdot }$, or less probably, $H_{2}O_{2}$. In this case, however, the catalytic generation of ROS is not related to the conversion of the structure of functional groups, and the generation of ROS is not limited in time—hence the greater effectiveness in eliminating bacteria [65,66]. As for layers with a functional group concentration of 1.25% (MSTF-Cu1.25%), their lower effectiveness is associated with a smaller number of catalytic centres (it is twice lower than for MSTF-Cu2.5%) and, consequently, lower catalytic activity of the material. The exact mechanism of ROS generation by the materials we describe is a very broad issue and is the subject of another research paper (article in preparation). Here, we limit ourselves to presenting the properties of the material.

Comparing the results with reference antibiotics, it can be concluded that the effectiveness of the tested materials is comparable to that of antibiotics but slightly lower. However, it should be remembered that the dry matter content of the material in the suspension used for the MIC tests is several times lower than that of antibiotics. It is not possible to carry out a quantitative analysis here, but it is safe to say that the antibacterial activity of the tested layers is significantly stronger than that of antibiotics.

**Figure 8:**
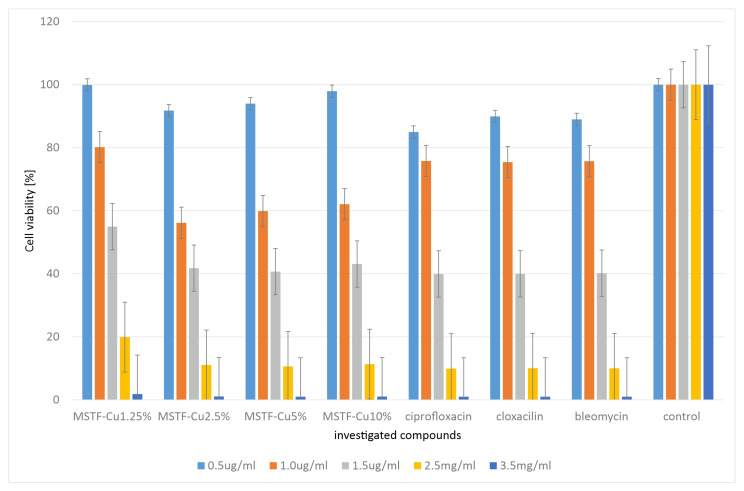
Measurement of cell viability (%) in the mouse embryonic fibroblast cell line (BALB/c3T3) after 24 h of incubation with of investigated samples: mesoporous silica thin films containing various concentrations of propyl-copper-phosphonate functional units inside pores in comparison to standard antibiotics: ciprofloxacin, cloxacillin, and bleomycin.

In order to check cytotoxicity, the presented materials (this time in powder form, as justified in Section 3.2) were subjected to MTT tests carried out using representative antibiotics: ciprofloxacin, bleomycin, and cloxacillin, as shown in Figure 8.

The results obtained indicate that the cytotoxicity of the tested compounds is comparable to that observed for the reference antibiotics. However, the cytotoxicity of samples containing 2.5 and 5% functional groups is slightly higher than that of materials containing 1.25 and 10% propyl-copper-phosphonate groups. The explanation for this is identical to that for the MIC studies. However, it should be emphasised that given the results obtained, the safety of the layers we propose should not be lower than that of antibiotics.

**Figure 9:**
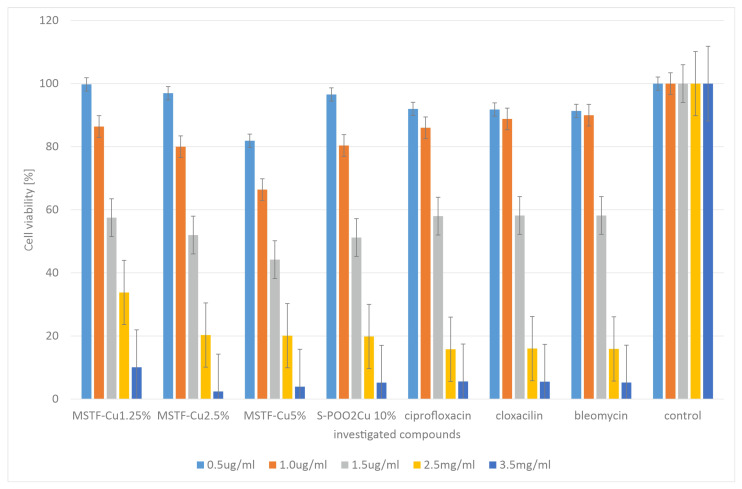
Measurement of cell viability (%) in the HeLa cell line after 24 h of incubation with of investigated samples: mesoporous silica thin films containing various concentrations of propyl-copper-phosphonate functional units inside pores, in comparison to standard antibiotics: ciprofloxacin, cloxacillin, and bleomycin.

We obtained similar results when testing the viability of HeLa cells (Figure 9).

Also, in this case, the results obtained indicate that the cytotoxicity of the tested compounds is comparable to that observed for the reference antibiotics. In this case, however, the material containing 5% active groups had the most cytotoxic effect. This may suggest that this concentration is optimal in terms of the efficiency of catalytic ROS generation. However, again in this case, we do not observe significantly higher cytotoxicity than for the reference antibiotics. At the same time, it should be emphasised here that the antibacterial mechanism associated with the generation of ROS is the only plausible one, since, as the study showed, no copper ions are released from the material. Furthermore, the surface physicochemical properties (SFE values) are similar for all the materials, so they are not the source of variation in terms of antimicrobial activity.

To sum up the microbiological tests, we can conclude that we have obtained a material with a high level of antibacterial activity, which at the same time is highly likely to remain safe for human cells.

## 3. Materials and Methods

### 3.1. Samples Synthesis

For the synthesis of propyl-copper-phosphonate functionalised mesoporous silica thin films, we used the combination of the evaporation-induced self-assembly (EISA) method with co-condensation of the silica precursor and the functional group precursor. By modifying the molar ratios between the precursors, it was possible to obtain materials containing different concentrations of functional groups. The concentrations were defined as the molar ratios of functional groups to SiO_2_ groups. Materials with functional group concentrations of 1.25%, 2.5%, 5% and 10% were prepared.

Reagents used in all procedures were at the highest possible purity. Triblock copolymer Pluronic P123 (EO_20_PO_70_EO_20_, where EO is polyethylene oxide and PO is polypropylene oxide), tetraethyl orthosilicate (TEOS), phosphonate propyl dietyltriethoxysilane (PPTES), bromotrimethylsilane (BrTMS), chlorotrimethylsilane (ClTMS) and copper acetylacetonate (Cu(acac)_2_)were purchased from Sigma-Aldrich Ltd. (Darmstadt, Germany).

The procedure of sol solution preparation for the dip-coating method involves five consecutive stages. The first includes a solution of ethanol and water in a weight ratio of 2.5:2 preparation, which was followed by adjusting the pH to 1.25 by adding HCl. Then, to this solution (5 mL), a 90 mmol mixture of tetraethyl orthosilicate (TEOS, Sigma-Aldrich) and phosphonate propyl diethyl triethoxysilane (PPTES, Sigma-Aldrich) were added in four different molar proportions (determining the molar concentration of functional groups in the target material), as shown in Table 3. These proportions directly determine the molar concentration of phosphonate functional groups towards SiO_2_ groups in the final material. TEOS acts as the precursor of the SiO_2_ network, while PPTES introduces propyl phosphonate groups into the silica matrix. Since PPTES also contains a silicon atom, its contribution to the total silica structure must be taken into account when calculating the density of functionalisation. For example, in a TEOS:PPTES molar ratio of 9:1, there is one phosphonate group per ten silicon atoms, which corresponds to a functionalisation level of 10% (in molar). By adjusting this ratio, we precisely control the number of anchoring groups available for subsequent copper complexation.

The obtained solution was continuously stirred for 2 h. Simultaneously, a second solution with a surfactant was prepared. This solution consists of 70 mL of a 3 mM ethanol solution of triblock copolymer Pluronic P123 (Sigma-Aldrich) prepared in a separate container and continuously stirred for 2 h. After two hours of mixing, both solutions were combined, and the obtained sol was stirred for another 3 h. The final stage was to add 4 g of HCl water solution with pH 1.25 to the final solution and leave it for aging for 2 h. The solution prepared in this way was used to deposit thin layers via the dip-coating method. In this method, the substrate on which the thin film is deposited is immersed in the solution and then withdrawn at an appropriate speed, resulting in a uniform coating of the substrate. In this case, glass substrates were used. The dip-coating method described here requires a closed-chamber dip-coater with fixed temperature and humidity conditions. The thin layers discussed here were prepared at a temperature of 22 °C and relative humidity of 75%. To obtain thin layers with a thickness of approximately 100 nm, the withdrawal speed was set at 2.5 mm/s. To obtain high-quality layers, substrates after withdrawal were left in the chamber for proper thin film formation for 20 min. To obtain a fully formed and stable thin layer, it is necessary to age it for at least 12 h at 100 °C. After aging, ethanol extraction of the organic skeleton is performed. In this stage, mesoporous thin films with precursor functional groups have to be transformed to silica with propyl-copper-phosphonate groups. It requires three more processes: sililation, hydrolysis and the introduction of metal ions. During sililation, silica thin films were immersed in 221 mM chlorotrimethylsilane (ClTMS) toluene solution for 12 h. The hydrolysis involved the immersion of thin films in a 1M solution of HCl for one hour. The introduction of copper ions to the silica matrix was performed by immersing the samples into a saturated solution of Cu(acac)_2_ in tetrahydrofuran for 12 h. Each procedure was followed by washing and drying under vacuum at 100 °C.

### 3.2. Characterisation Methods

Transmission electron microscopy (TEM) imaging was carried out using an FEI Tecnai G2 20 X-TWIN electron microscope (FEI Company, Eindhoven, The Netherlands) equipped with a LaB_6_ emission source and FEI Eagle 2 K CCD camera.

The surface morphology of the samples was examined using a Tescan Vega 3 scanning electron microscope (SEM)(Tescan Orsay Holding, Brno, Czech Republic). All images were recorded at 15 kV using the SE detector with multiple areas selected randomly for observation. Energy-dispersive X-ray spectroscopy (EDS) elemental analysis was performed using the same microscope equipped with a Bruker QUANTAX EDS detector (Bruker Nano GmbH, Berlin, Germany). Sample preparation involved mechanically removing thin layers of the silica coating from the glass substrate and placing them on carbon adhesive tape to eliminate substrate interference in the elemental analysis.

The molecular structure of samples was characterised using confocal Raman microscope WITec alpha 300R (WITec GmbH, Ulm, Germany) operatingwith a laser λ = 532 nm, a high-performance low-dark current CCD camera ANDOR iVac DR-316B-LDC-DD-35B (Andor Technology Ltd., Belfast, UK), and UHTS300 SMFC VIS-NIR spectrometerwith a focal length of 300 mm and grating 600 grooves/mm BLZ = 500 nm. The spectra were accumulated at a laser power of 20 mW over 50 scans with an integration time of 3 s using a Zeiss EC Epiplan-NeofluarDic (Carl Zeiss Microscopy GmbH, Jena, Germany) 100×/0.9 objective. All samples were scraped off the substrates to eliminate strong signals from the glass.

ROS generation was detected using a fluorescent CellROX^TM^ Green Reagent (Invitrogen, Life Technologies, Carlsbad, CA, USA). The dye is weakly fluorescent in its reduced state and exhibits bright green fluorescence upon oxidation by reactive oxygen species (ROS) with absorption/emission maxima of ∼485/520 nm. CellROX^TM^ Green Reagent was dissolved in phosphate-buffered saline (PBS) at a volume ratio of 1:4. Grains of mesoporous silica were placed on the glass support, and a drop of CellROX^TM^ Green Reagent solution was subsequently applied on top. A fluorescence microscope (Leica DMi8, Heidelberg, Germany) equipped with 40× objective was used to confirm the presence of a green fluorescence signal as well as to collect bright-field images of the silica grains. Microscopic images were collected immediately upon CellROX^TM^ Green reagent treatment.

The contact angle (water and glycerine CA) was measured using a droplet shape analyser (JC2000D Contact Angle Tester, Shanghai Zhongchen Digital Technic Apparatus Co., Ltd., Shanghai, China)under ambient conditions at room temperature and humidity. In each drop, we used 5 μL of deionised water and glycerine. The contact angle was measured immediately after dropping the liquid. For all samples, we checked the change in contact angle over time by taking an additional measurement 30 s after dropping. In the case of the tested samples, the contact angle remained constant over time. To calculate the surface free energy (SFE), we used the Owens–Wendt model, which involves two test liquids [67]. In our case, we used distilled water and glycerin as reliable for such measurements [68].

Copper release tests were conducted to determine whether there is direct interaction between copper released from the material and the microorganisms under test. The test consisted of immersing the test material in the form of a thin layer on glass, cut to a size of 1 × 2 cm, immersing it in 5 mL of normal saline solution, and leaving it at 37 °C for ten days. During this time, the samples were mixed twice a day. Each sample was prepared in four replicates. The resulting liquid was poured off the sample after 24 h, 48 h, 6 days, and 10 days, and the copper content was determined by atomic absorption spectroscopy (AAS) with the use of a Shimadzu model AA-680 atomic absorption spectrometer (Shimadzu Corporation, Kyoto, Japan)having a hollow cathode lamp and a deuterium background with an air–acetylene flame. The lamp current was set to 6 mA. Measurements were carried out in the integrated absorbance mode at 324.8 nm, using a slit width of 0.7 nm

Microbiological tests were carried out on both Gram-positive and Gram-negative bacteria. The reference bacterial strains of *Escherichia coli* (K12 ATCC 25404, R2 ATCC 39544, R3 ATCC 11775, and R4 ATCC 39543), *Staphylococcus aureus* strain (ATCC 23235), *Acinetobacter baumannii* (ATCC 17978), *Pseudomonas aeruginosa* (ATCC 15442), and *Enterobacter cloacae* (ATCC 49141) were obtained from LGC Standards U.K. and were used according to the recommendation of ISO 11133 [69]. These strains were used to test the antibacterial activity of the analysed compounds by determining the minimum inhibitory concentration (MIC).

MIC test results were estimated using a microtiter plate method with sterile 8- or 96-well plates [69,70,71,72]. In brief, we prepared solutions containing the tested material (MSTF-Cu1.25, MSTF-Cu2.5%, MSTF-Cu5%, and MSTF-Cu10%) scratched from 1 cm^2^ of the glass substrate and suspended in 100 μL of distilled water. Then, the 100 μL of analysed suspensions and the appropriate bacterial strains were added to the first row of the plate. Then, 25 μL of sterile tryptone soya broth (TSB) medium was added to the other wells, and serial dilutions were performed. Subsequently, 200 μL of inoculated TSB medium containing resazurin (0.02 mg/mL) as an indicator was added to all wells. TSB medium was inoculated with 10^6^ colony-forming units (CFU)/mL (approximately 0.5 McFarland units) of the bacterial strains. The plates were incubated at 37 °C for 24 h. Changes in colour from blue to pink or yellowish with turbidity were considered positive, and the lowest concentration at which no visible change in colour occurred was recorded as the MIC [71]. Each experiment was repeated at least three times. As a reference point for our materials, we used the standard antibiotics: ciprofloxacin, cloxacillin, and bleomycin. In this case, we obtained the starting dilution by dissolving 1 mg of the antibiotic in 1 mL of distilled water. The subsequent procedure was identical to that for the test materials.

The cytotoxic effects of the four tested compounds on BALB/c3T3 mouse fibroblast cells and HeLa were determined using the MTT assay after 24 h of incubation at five different concentrations (0.5, 1.0, 1.5, 2.5, and 3.5 μg/mL). The MTT test is based on the ability of the mitochondrial dehydrogenase enzymes to convert an orange, water-soluble tetrazolium salt (3-(4,5-dimethylthiazol-2-yl)-2,5-diphenyltetrazolium bromide) into an insoluble formazan, which is a dark blue product of the above reaction. After dissolving the formazan crystals in DMSO or isopropanol, a coloured solution was formed, the intensity of which is measured spectrophotometrically within the wavelength range of 492–570 nm. The amount of coloured reduced MTT is proportional to the oxidative activity of the cell’s mitochondria and, under strictly defined experimental conditions, to the number of metabolically active (living) cells in the population. We applied the standard MTT procedure [73,74]. The MTT tests were performed on a slightly different form of material than the tested thin layers. Due to the inability to weigh the appropriate amount of material when scraping, we used a powder form obtained in accordance with the procedure described in the study [52]. For the cytotoxicity tests, we assumed the least favourable case that the powder form of SBA-15 would be more cytotoxic than the layer. Therefore, we decided to use powder and generalise the results to thin layers.

Photos of culture plates used to obtain the results for the graphs are provided in the Supporting Information (Figures S2 and S3).

## 4. Conclusions

In this study, we present a novel type of nanostructured functional coating that effectively inhibits the growth of pathogenic microorganisms. This coating was engineered to continuously generate reactive oxygen species (ROS) in the presence of water or moisture without requiring any external activating agent.

The desired functionality was achieved through a nanocomposite structure composed of a mesoporous SBA-15 silica matrix and uniformly distributed propyl-copper-phosphonate functional groups. The copper phosphonate groups serve as catalysts for ROS generation, while the silica matrix ensures their uniform distribution, stores the generated ROS, and enables their controlled release over time.

The material was prepared in the form of thin layers on a glass substrate and tested for the intended structure and properties. The coatings were prepared in four variants, differing in the content of functional groups (from 1.25 to 10% by mole relative to SiO_2_ groups). Physicochemical studies showed that the material obtained was consistent with the assumptions. TEM microscopy showed the assumed structure of the silica matrix in the form of a material containing long, parallel 2D hexagonally arranged pores with diameters of approximately 5 nm. SEM microscopy combined with EDS elemental analysis showed the assumed chemical composition and homogeneous distribution of elements, which was the basis for concluding that the functional groups also have the assumed structure. Raman spectroscopy largely confirmed this, showing the presence of vibration modes from the assumed molecular structures. In addition, fluorescence microscopy explicitly showed that the material generates reactive oxygen species throughout its volume. Furthermore, observation of areas where the coating was damaged showed that ROS generation is local and closely related to the presence of a functional silica coating.

However, biological tests were the most important for assessing the material’s compliance with the assumptions. They showed that the material can be used as a coating to protect against microorganisms. Bacteriological tests on Gram-negative and Gram-positive strains showed the high antibacterial activity of the tested material. In each case (i.e., for materials containing all concentrations of functional groups), antibacterial activity was observed that was at least comparable to that of reference antibiotics. However, the activity of individual materials varied considerably. Namely, materials containing 2.5 and 5% functional groups were the most effective, while those containing 1.25% and 10% were significantly less effective.

This observation was related to the configuration of the functional groups as well as their quantity. For the material with the highest concentration of propyl-copper-phosphonate groups, they are positioned so close to each other that ROS catalysis associated with the conversion of their molecular structure is possible (the groups can interact with each other). For lower concentrations, the distance between the active groups is large enough that they cannot interact with each other, so conversion of their structure is not possible, and therefore, the catalysis mechanism is different, resulting in the generation of other types of ROS. Furthermore, this process is much faster and does not require regeneration of the catalytic centres. The material with the lowest concentration of functional groups, in turn, exhibits lower antimicrobial activity due to the reduced number of catalytic centres and, therefore, lower catalytic activity. However, a detailed analysis of the catalytic properties of the presented material is beyond the scope of this work and is the subject of a separate study.

Despite their antimicrobial activity, the presented layers remain relatively safe for cells. MTT tests conducted on BALB/c3T3 mouse fibroblast cells and HeLa cells showed that the material is no more cytotoxic than the reference antibiotics.

The results obtained confirm the high application potential of the presented material. Its broad-spectrum antibacterial activity, including low MIC values against strains such as *Staphylococcus*, indicates strong biocidal effects even at low concentrations. This is particularly important in healthcare settings, where surface coatings that limit the spread of harmful microorganisms are increasingly in demand. Importantly, the material maintains low cytotoxicity, making it a promising candidate for future biomedical applications, especially in the context of emerging infectious diseases.

## 5. Patents

The results of the work presented here are the subject of patent application P.451615.

## Figures and Tables

**Table 1:** Results of the energy-dispersive X-ray spectroscopy (EDS) elemental analysis of obtained samples: mesoporous silica thin films containing various concentrations of propyl-copper-phosphonate functional units inside pores. The silicon-to-phosphorus and phosphorus-to-copper ratios were measured separately to increase the accuracy of the measurement.

	Element Content (At. %)			
	Si:P		P:Cu	
Sample	Si	P	P	Cu
MSTF-Cu1.25%	98.9	1.1	48.0	52.0
MSTF-Cu2.5%	97.6	2.4	48.9	51.1
MSTF-Cu5%	95.0	5.0	50.8	49.2
MSTF-Cu10%	91.2	8.8	56.7	43.3

**Table 2:** Contact angles (CA) and calculated surface free energy ($\gamma_{S}$) for investigated samples: mesoporous silica thin films containing various concentrations of propyl-copper-phosphonate functional units inside pores.

Material	H_2_O CA [°]	Glycerin CA [°]	$\gamma_{S}$ [mJ/m^2^]
MSTF-Cu1.25%	63.22	58.92	53.74612
MSTF-Cu2.5%	66.15	58.57	46.92306
MSTF-Cu5%	68.05	58.07	57.47949
MSTF-Cu10%	68.55	62.97	35.84863

**Table 3:** TEOS to PPTES ratios used in co-condensation procedure.

Sample	Molar Ratio TEOS:PPTES
MSTF-Cu1.25%	79:1
MSTF-Cu2.5%	39:1
MSTF-Cu5%	19:1
MSTF-Cu10%	9:1

## Data Availability

Source data are available on request.

## Supplementary Materials

The following supporting information can be downloaded at https://www.mdpi.com/article/10.3390/ijms26157154/s1.

## Abbreviations

The following abbreviations are used in this manuscript:

TEM	Transmission electron microscope
SEM	Scanning electron microscope
EDS	Energy-Dispersive X-ray Spectroscopy
TEOS	Tetraethoxysilane
PPTES	Phosphonate propyl dietyltriethoxysilane
TMS	Trimethylsilyl groups
