# Supplementary material for: Functionalised Mesoporous Silica Thin Films as ROS-Generating Antimicrobial Coatings
Source: Int J Mol Sci. 2025 Jul 24;26(15):7154. doi: 10.3390/ijms26157154 (PMC12345941; doi:10.3390/ijms26157154)
Supplement: Supplementary file 1 [file ijms-26-07154-s001.zip › ijms-3774133-supplementary.pdf]

Article

Supporting Information for: Functionalized mesoporous silica thin films as ROS-generating antimicrobial coatings

Magdalena Laskowska [1]*, Paweł Kowalczyk [2], Agnieszka Karczmarska [1], Katarzyna Pogoda [1], Maciej Zubko [3], and Łukasz Laskowski [1]*

[1] Institute of Nuclear Physics Polish Academy of Sciences, PL-31342 Krakow, Poland
[2] Institute of Materials Engineering, University of Silesia in Katowice, 75 Pułku Piechoty 1A St., 41-500 Chorzów, Poland
[3] Faculty of Production Engineering and Materials Technology, Częstochowa University of Technology, 42-201 Częstochowa, Poland
* Correspondence: magdalena.laskowska@ifj.edu.pl, lukasz.laskowski@ifj.edu.pl

1. Contact angle measurements

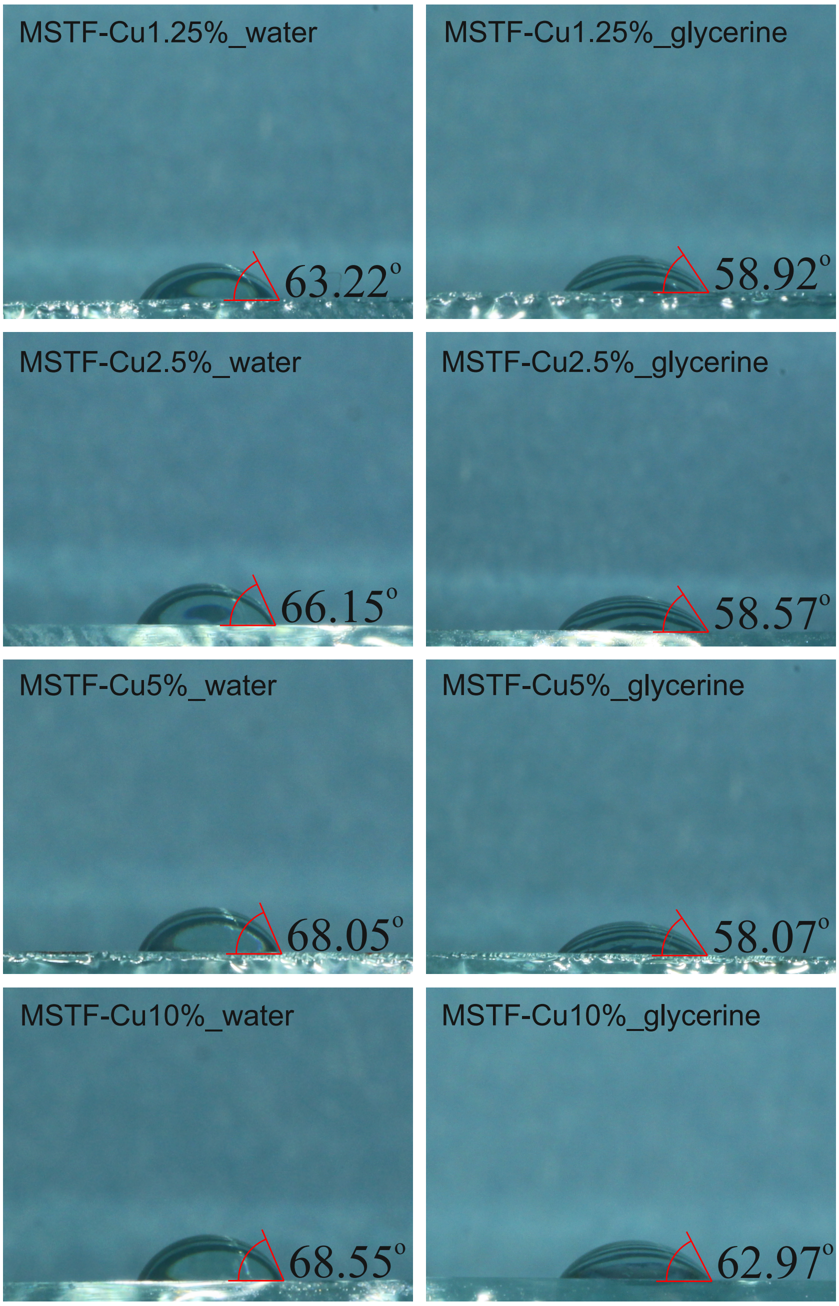

Figure S1. Source images from contact angle measurements for investigated samples: mesoporous silica thin films containing various concentrations of propyl copper phosphate functional units inside pores, using distilled water and glycerine. The images indicate the contact angles.

2. pictures of the growing plates used for experiments

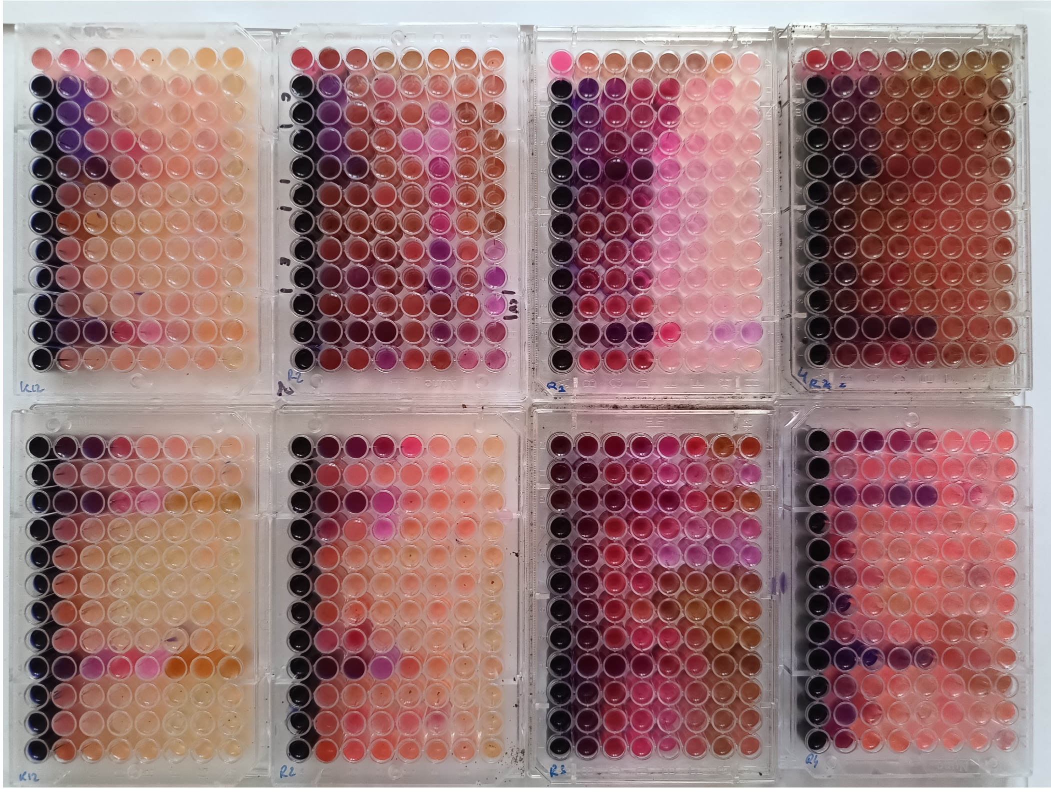

Figure S2. Minimum inhibitory concentration (MIC) of investigated samples: mesoporous silica thin films containing various concentrations of propyl copper phosphate functional units inside pores, in comparison to standard antibiotics: ciprofloxacin, cloxacillin, and bleomycin. Pictures of growing plates.

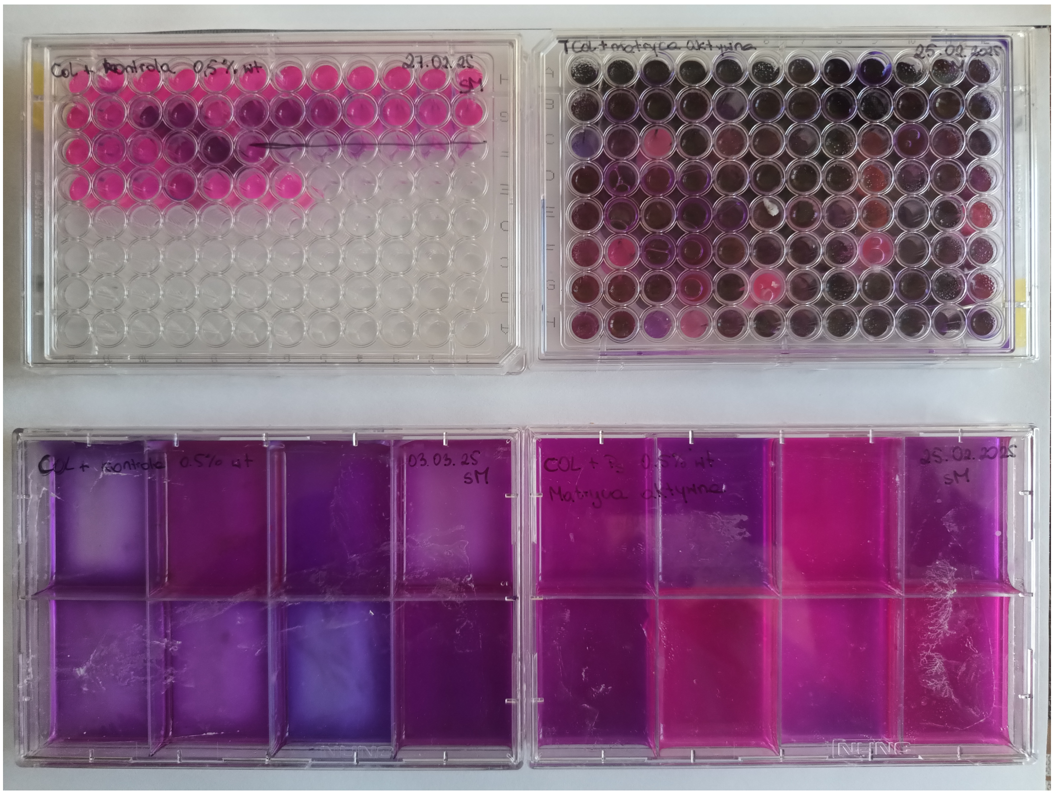

Figure S3. Tests of cell viability in the mouse embryonic fibroblast cell line (BALB/c3T3) and HeLa cell line after 24 h of incubation with of investigated samples: mesoporous silica thin films containing various concentrations of propyl copper phosphate functional units inside pores, in comparison to standard antibiotics: ciprofloxacin, cloxacillin, and bleomycin. Pictures of growing plates.
